# Insecticide Resistance and Target-Site Mutations *kdr*, *N1575Y*, and *Ace-1* in *Anopheles gambiae s.l.* Populations in a Low-Malaria-Transmission Zone in the Sudanian Region of Senegal

**DOI:** 10.3390/genes15101331

**Published:** 2024-10-16

**Authors:** Assiyatou Gueye, El Hadji Malick Ngom, Baye Bado Ndoye, Mamadou Lamine Dione, Babacar Diouf, El Hadji Ndiaye, Faty Amadou Sy, Marième Guèye, Makhtar Niang, Diawo Diallo, Mawlouth Diallo, Ibrahima Dia

**Affiliations:** 1 Pôle de Zoologie Médicale, Institut Pasteur de Dakar, Dakar BP 220, Senegal; assiyatou.gueye@pasteur.sn (A.G.); elhadjimalick.ngom@pasteur.sn (E.H.M.N.); bayebado@gmail.com (B.B.N.); alaminedione94@gmail.com (M.L.D.); babacar.diouf2@pasteur.sn (B.D.); elhadji.ndiaye@pasteur.sn (E.H.N.); fatyamadou.sy@pasteur.sn (F.A.S.); mariamegueye02@gmail.com (M.G.); diawo.diallo@pasteur.sn (D.D.); mawlouth.diallo@pasteur.sn (M.D.); 2 Pôle Immunophysiopathologie et Maladies Infectieuses, Institut Pasteur de Dakar, Dakar BP 220, Senegal; makhtar.niang@pasteur.sn

**Keywords:** malaria vectors, resistance mechanisms, target-site mutations, *anophelines*, Senegal

## Abstract

**Background/Objectives:** Significant progress in malaria control has been achieved through long-lasting insecticidal nets (LLINs) and indoor residual spraying (IRS), raising hopes for malaria elimination. However, emerging insecticide resistance threatens these gains. This study assessed the susceptibility of *Anopheles gambiae s.l.* populations to public health insecticides, examined the frequencies of *kdr*, *Ace-1*, and *N1575Y* mutations, and explored their associations with phenotypic resistance in Dielmo and Ndiop, Senegal. **Methods**: *Anopheles* larvae collected between September and December 2022 were reared to adulthood. Adult mosquitoes were exposed to discriminating concentrations of various insecticides following WHO guidelines. Knockdown times (KDT50 and KDT95) for pyrethroids were calculated using the Probit model. RT-qPCR detected target-site mutations (*kdr*: *L1014F* and *L1014S*, *Ace-1*, *N1575Y*) and assessed correlations with phenotypic resistance. Species-specific PCR identified species within the *An. gambiae* complex. **Results/Conclusions**: The populations of Dielmo and Ndiop showed susceptibility to pirimiphos-methyl and bendiocarb, with no *Ace-1* mutation detected. Resistance to DDT and pyrethroids was observed. The knockdown times indicated that alphacypermethrin and lambdacyhalothrin were more effective than permethrin and deltamethrin. The *L1014F* allele was widespread, while *L1014S* was absent in Ndiop and low in Dielmo. The *N1575Y* mutation occurred only in populations with *L1014F*. The *L1014S* mutation was significantly associated with resistance to lambdacyhalothrin in both villages and to deltamethrin in Ndiop.

## 1. Introduction

Substantial progress in malaria control over recent decades has led to a notable decline in both incidence and mortality rates, fostering realistic optimism regarding the potential for disease elimination [1]. This success is largely attributed to the widespread implementation of strategies such as long-lasting insecticidal nets (LLINs) and indoor residual spraying (IRS), which rely heavily on the efficacy of insecticides to reduce vector populations and interrupt malaria transmission. Despite these achievements, the emergence and spread of insecticide resistance among malaria vectors present a major challenge to sustaining these control efforts [2,3].

While efforts to explore new classes and combinations of insecticides are ongoing, pyrethroids remain the primary insecticides for treating mosquito nets due to their mosquito-repelling properties and relatively low toxicity to mammals [4]. However, extensive pyrethroid use has led to a marked decrease in their effectiveness, highlighting the urgent need for alternative solutions. Resistance is driven by the selection of resistant alleles, giving certain mosquitoes a survival advantage in environments with high insecticide levels [5], thereby posing a significant threat to future malaria control efforts [6].

Resistance to pyrethroids, DDT, and carbamates has been documented across Africa among *Anopheles* populations [7,8]. These mosquitoes have developed various survival mechanisms against lethal insecticide doses [9]. These resistance mechanisms include genetic mutations that alter insecticide target sites and enhanced metabolic detoxification pathways.

Knockdown resistance (*kdr*) mutations, such as *L1014F* and *L1014S*, are well-documented contributors to resistance against pyrethroids and DDT and are prevalent in regions across Africa, including West and East Africa [10,11,12]. These mutations disrupt the sodium channel proteins targeted by pyrethroids, leading to cross-resistance. Other target-site mutations, such as *G119S* in the *Ace-1* gene, are associated with resistance to carbamates and organophosphates in *An. gambiae s.l.* populations [13,14]. Additionally, the *N1575Y* mutation, located in intracytoplasmic loop linking domains III and IV of sodium channels, has been identified in populations of *An. gambiae s.l.* carrying the *L1014F* mutation, further complicating resistance profiles [15,16].

In Senegal, particularly within the Sudanian zone, the dynamics of insecticide resistance and the prevalence of these mutations are not fully understood. Previous studies have highlighted significant resistance to DDT and pyrethroids, but comprehensive data on specific mutations and their roles in resistance remain limited [17,18]. This knowledge gap is critical, as understanding resistance mechanisms in local populations is essential for optimizing vector control strategies. More specifically, in Dielmo village, prior investigations indicated the absence of the *kdr L1014S* mutation in pyrethroid-resistant *An. gambiae* populations, with only *Anopheles arabiensis* and *Anopheles coluzzii* populations exhibiting the *L1014F* and *L1014S* alleles [19]. This suggests that other resistance mechanisms, potentially involving different target sites or metabolic pathways, may be at play in these populations. Additionally, the absence of comprehensive data on the *N1575Y* and *Ace-1* mutations in the Sudanian zone further underscores the need for detailed studies.

Given these evolving resistance dynamics, this study aims to (1) evaluate the current insecticide susceptibility profiles of *Anopheles* populations from Dielmo and Ndiop to commonly used insecticides, including organochlorines, organophosphates, carbamates, and pyrethroids; (2) assess the frequency and distribution of the key target-site mutations *L1014F*, *L1014S*, *N1575Y*, and *G119S*; and (3) identify any associations between these genetic markers and the observed resistance phenotypes.

## 2. Materials and Methods

### 2.1. Study Area

**Figure 1:**
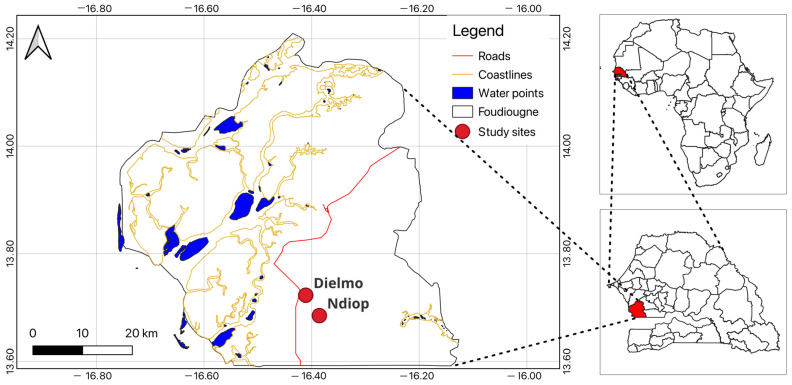
Map of the study area.

This study was conducted in two locations in the Sudanian zone of Senegal: Dielmo (13°43′22.2″ N, 16°24′40.1″ W) and Ndiop (13°41′12.8″ N, 16°23′3.2″ W) (Figure 1). Longitudinal studies on malaria determinants have been ongoing in these locations since 1990 and 1993, respectively [20]. Over the years, the epidemiology of malaria has changed significantly, leading to considerations of disease elimination in this area [20,21]. However, a resurgence of malaria cases has been observed in both villages [22,23].

In Dielmo, a small river (Nema) flows year-round, ensuring the constant presence of vectors [24]. In contrast, malaria transmission in Ndiop is seasonal, with vector breeding sites dependent on rainfall [25].

The main economic activity in both villages is agriculture, focused on groundnuts, cashew nuts, mangoes, and vegetables. Consequently, commercial pesticides, including organophosphates, organochlorines, and pyrethroids, are commonly used.

Malaria transmission in Dielmo and Ndiop is primarily maintained by species from the *An. gambiae* complex, including *An. arabiensis*, *An. gambiae*, and *An. coluzzii,* as well as *An. funestus* [26].

The two villages were selected based on the persistence of malaria transmission despite the control measures implemented.

### 2.2. Mosquito Collection and Rearing

*Anopheles* mosquito larvae were collected over two consecutive days each month from various breeding sites between September and December 2022 from 8:00 am to noon using the methods outlined by Service [27]. Visits were conducted to each site, covering different types of breeding habitats such as river banks, ponds, water tanks, and rain puddles. Upon detecting productive sites, *Anopheles* larvae and pupae were collected and sorted into bins according to their habitat type and location. These specimens were transported to the laboratory and reared under standard insectary conditions of 70% to 80% relative humidity and 27 °C to 28 °C ambient temperature. Larvae were fed with Tetramin^®^ baby fish food, and emerging adult mosquitoes were provided with a 10% glucose solution before being exposed to insecticides.

### 2.3. WHO Insecticide Susceptibility Bioassays

Susceptibility assays were conducted following the World Health Organization protocol [28], using three-to-five-day-old unfed female mosquitoes whenever available. Seven insecticides were tested: four pyrethroids (deltamethrin 0.05%, lambdacyhalothrin 0.05%, alphacypermethrin 0.05%, and permethrin 0.75%), one organochlorine (DDT 4%), one organophosphate (pirimiphos-methyl 0.25%), and one carbamate (bendiocarb 0.1%).

Batches of 20 to 26 females were exposed to insecticide-impregnated papers for one hour. For each test, two batches, each consisting of 20 to 25 females, were exposed to untreated papers as controls. The number of knocked-down mosquitoes was recorded after 10, 15, 20, 30, 40, 50, and 60 min of exposure. Following the one-hour exposure period, the mosquitoes were transferred to observation tubes and provided with a 10% glucose solution. The mortality rates of the females were assessed 24 h later.

### 2.4. Mosquito Species Identification

All *An. gambiae s.l.* specimens exposed to insecticides underwent species-level identification using a binocular microscope and the morphological identification key of Robert et al. [29]. They were then preserved in Eppendorf tubes containing silica gel and cotton for further analysis.

### 2.5. Molecular Identification and Genotyping of kdr and Ace-1 Mutations by PCR

Among the tested females, for each month, up to 60 individuals, with 30 per status (dead or alive), were selected for each insecticide for further molecular identification. DNA extraction was performed using the cetyl-trimethyl-ammonium-bromide (CTAB) 2% method [30]. Each specimen was ground using a sterile pestle in 200 μL of CTAB 2%. The tubes were then incubated in a dry bath at 65 °C for 5 min. After cooling, 200 μL of chloroform was added to each tube, mixed thoroughly, and centrifuged at 12,000 rpm for 5 min. The supernatant was transferred to a new tube, and 200 µL of isopropanol was added. The tubes were centrifuged at 12,000 rpm for 15 min, and the supernatant was discarded. The DNA pellet was washed with 200 μL of 70% ethanol, centrifuged at 12,000 rpm for 5 min, air-dried, resuspended in 40 μL of sterile pure water, and stored at −20 °C after overnight incubation at room temperature.

The extracted DNA was used for molecular identification of species within the *An. gambiae* complex, following the techniques described by Scott et al. [31] and Fanello et al. [32]. The quantitative Polymerase Chain Reaction (qPCR) technique with the Luna Universal Probe assay from New England Biolabs was employed to detect the *L1014F*, *L1014S*, *N1575Y*, and *Ace-1* mutations. Genotyping followed protocols by Bass et al. [33] for *L1014F* (*kdr-West*) and *L1014S* (*kdr-East*), Jones and colleagues [15] for the *N1575Y* mutation, and Bass and colleagues [34] for the *Ace-1* mutation.

### 2.6. Data Analysis

Mosquito mortality was evaluated at 24 h post-exposure, with validity and susceptibility status determined according to WHO criteria [28]. Tests were considered valid if control mortality was below 5%, required correction using the Abbott formula [35] if between 5% and 20%, and invalid if above 20%.

Population resistance classification was based on mortality rates: confirmed resistance (≤90% mortality), possible resistance (90% and 98%), and susceptibility (≥98%). Mortality rates and allele frequencies were estimated for each population, and data analysis was performed with a 95% confidence interval. Knockdown times (KDT_50_ and KDT_95_) were calculated using the Probit model with a 95% confidence interval using the *BioRssay* package [36]. Comparisons between *Anopheles* species abundance and mortality rates were performed using the Chi-squared test or Fisher’s exact test, with a significance threshold of 0.05.

Comparisons of *kdr* mutation frequencies were conducted based on survival or mortality status following the tests using the *fmsb* package available in the R software (https://cran.r-project.org/web/packages/fmsb/index.html, accessed on 13 September 2023). The relationship between *kdr* mutations (*L1014F* and *L1014S*) and pyrethroid resistance was analyzed using allelic association analysis. Odds ratios (ORs) and 95% confidence intervals (CIs) were calculated to determine the strength of association between *kdr* mutations and resistance, with statistical significance determined using a *p*-value of <0.05.

All statistical analyses and graphs were created using R software version 4.3.2. [37].

## 3. Results

### 3.1. Insecticide Resistance Profile

Insecticide susceptibility tests on *Anopheles* populations from Dielmo and Ndiop are summarized in Table 1. In all bioassays, the control tests showed mortality rates below 5%, so no corrections were required in the tested sample data. A total of 2139 specimens were exposed to the WHO-recommended discriminating concentrations. *Anopheles* mosquitoes showed complete susceptibility to pirimiphos-methyl and bendiocarb at both sites. However, resistance to DDT and pyrethroids was observed, with mortality rates ranging from 31% to 88% (Table 1).

Mortality rates with lambdacyhalothrin decreased over time, from September to December in Dielmo and from September to October in Ndiop (Table 1). This decrease was statistically significant (*p* < 0.05) at both sites. Conversely, a significant increase in mortality rates with deltamethrin was observed in Dielmo between November and December (Table 1).

### 3.2. Knockdown Effects and Dynamics of Pyrethroids

Table 2 presents the mean knockdown times (KDT_50_ and KDT_95_) for 50% and 95% of mosquitoes, respectively. In both sites, deltamethrin had the shortest KDT_50_ times, at 25 min, followed by permethrin at 28 min for Ndiop and 31 min for Dielmo. Similar profiles were observed for KDT_95_ (Table 2).

**Figure 2:**
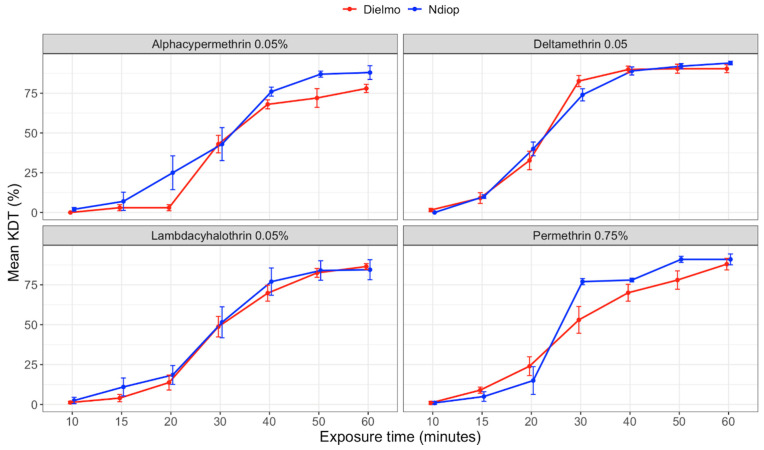
Variations in mean pyrethroid KDT rates according to exposure times for *An. gambiae s.l.* populations from Dielmo and Ndiop to different insecticides from September to December 2022.

The dynamics of pyrethroid action showed that resistance was associated with a low insecticide action dynamic (Figure 2). None of the four tested pyrethroids achieved 100% knockdown after 60 min of exposure. The highest mean knockdown (KD) values were observed with deltamethrin in Dielmo at 50 and 60 min (90.4%) and in Ndiop at 60 min (94%), as well as with permethrin at 88% in Dielmo and 91% in Ndiop after 60 min. Conversely, alphacypermethrin showed the lowest knockdown rates.

### 3.3. Species Composition

Species-specific PCR analysis on 729 mosquitoes from Dielmo and 412 mosquitoes from Ndiop (out of the 2139 specimens exposed to insecticides) identified *An. arabiensis* as the predominant species, comprising 84.9% in Dielmo and 83.5% in Ndiop. *An. gambiae* was the second most abundant species, while *An. coluzzii* was less common, accounting for 3.6% in Dielmo and 3.4% in Ndiop. Hybrids between *An. gambiae* and *An. coluzzii* were rare, with only three individuals (0.4%) in Dielmo and five individuals (1.2%) in Ndiop (Table 3). The species proportions were similar in both sites, with no statistically significant difference (*p* > 0.05).

### 3.4. Genotypic and Allelic Frequencies of the L1014F and L1014S Mutations

All six genotypes at the 1014 locus were identified in *An. arabiensis* populations, while four were found in *An. coluzzii* and three in *An. gambiae* (Table 4). The homozygous wild-type LL and heterozygous LS genotypes were most common in *An. arabiensis,* with frequencies of 37.3% and 29.3% in Dielmo and 32% and 34% in Ndiop, respectively. In these populations, 32.6% (106/325) in Dielmo and 31.4% (60/191) in Ndiop were the homozygous susceptible LL genotype (Table 4). Heterozygotes resistant to the *L1014F* mutation constituted 31.1% (101/325) in Dielmo and 29.8% (57/191) in Ndiop, while resistance to the *L1014S* mutation was observed in 24% (78/325) and 26.2% (50/191), respectively.

The *L1014F* allele was present in all populations from both sites, whereas the *L1014S* allele was absent in *An. gambiae* females from Ndiop and found at a low frequency (1%) in Dielmo (Table 4). Among *An. coluzzii* and *An. gambiae*, the *L1014F* allele was more frequent than the *L1014S* allele, while in *An. arabiensis*, the 1014S allele was predominant. No significant difference in *L1014F* allele frequencies was observed between the two sites for each species.

### 3.5. Genotypic and Allelic Frequencies of the N1575Y and Ace-1 Mutations

Overall, the *N1575Y* mutation was absent in *An. arabiensis* and *An. coluzzii* at both sites and present at a low frequency in *An. gambiae* from Dielmo (2% in two specimens). The homozygous resistant genotype YY was not observed in any species at either site.

In *An*. *arabiensis*, the wild-type N allele was present at a frequency of 100% in both Dielmo and Ndiop. Similarly, *An. coluzzii* populations also showed a 100% frequency at both sites. For *An. gambiae*, the N allele frequency was 98% in Dielmo and 100% in Ndiop, with the Y allele detected only in Dielmo (Table 5).

The *Ace-1* mutation was investigated in 241 specimens of *An. gambiae s.l.* from Dielmo and Ndiop. All these specimens were susceptible to bendiocarb and pirimiphos-methyl. The *Ace-1* mutation was not identified in any of the tested specimens.

### 3.6. Association between the L1014F and L1014S Mutations and Pyrethroid Resistance

The analysis of the *L1014F* and *L1014S* mutations in relation to pyrethroid resistance in *An. gambiae s.l.* populations from Dielmo and Ndiop revealed significant associations for certain combinations of mutations and insecticides.

For the *L1014F* mutation, no significant association with resistance to the tested pyrethroids—alphacypermethrin, deltamethrin, lambdacyhalothrin, and permethrin—was observed at either sites. The odds ratios (ORs) for *L1014F* varied but did not indicate a strong relationship with insecticide resistance (Table 6), suggesting that this mutation is not a major determinant of pyrethroid resistance in these populations.

In contrast, the *L1014S* mutation was significantly associated with resistance to some pyrethroids. In Ndiop, significant correlations were found between the *L1014S* mutation and resistance to lambdacyhalothrin (*p* = 0.01) and deltamethrin (*p* = 0.03). In Dielmo, this mutation was notably linked to resistance to lambdacyhalothrin (*p* = 0.0002). However, no significant correlation was observed between the *L1014S* mutation and resistance to alphacypermethrin and permethrin at either site or to deltamethrin in Dielmo (*p* > 0.05) (Table 7). The odds ratios indicated that individuals with the *L1014S* mutation were more likely to exhibit resistance to these pyrethroids.

## 4. Discussion

This study aimed to evaluate the insecticide resistance profiles of species within the *An. gambiae* complex and to determine the frequencies of the *kdr* (*L1014F* and *L1014S*), *Ace-1*, and *N1575Y* mutations in the Sudanian zone of Senegal. Species-specific PCR analysis identified *An. arabiensis* as the predominant species at both study sites, followed by *An. gambiae* and *An. coluzzii*. This composition is consistent with other studies in West Africa, where *An. arabiensis* is frequently reported as the dominant vector species in areas with significant agricultural activity [17,38,39]. The similarity in species proportions between the sites suggests a stable vector community structure, crucial for developing targeted vector control interventions. This stability aligns with the adaptability of *An. arabiensis* to arid environments and its preference for habitats such as riverbanks and ponds, which are primary breeding sites in our study area [19].

Insecticide susceptibility tests revealed resistance to DDT and pyrethroids, with mortality rates ranging from 31% to 88%. These findings are consistent with numerous studies across Africa documenting widespread resistance to these insecticides in *Anopheles* populations [28]. Similar results have been observed across different regions of Senegal [11,17,18]. This cross-resistance to pyrethroids and DDT may be attributed to historical insecticide use in vector control and agricultural practices [18,40,41,42]. Resistance to pyrethroids is particularly concerning given their widespread use in long-lasting insecticidal nets (LLINs) and indoor residual spraying (IRS), cornerstone strategies for malaria vector control [28]. The observed decrease in mortality rates with lambdacyhalothrin over time in both Dielmo and Ndiop highlights the dynamic nature of insecticide resistance and suggests that continuous monitoring and timely adaptation of vector control strategies are essential. This trend mirrors findings from other regions, where prolonged use of pyrethroids has led to significant increases in resistant mosquito populations [11,17,18]. The concurrent use of pyrethroids in insecticide-treated bed nets [21] and the intense agricultural use of pyrethroids likely contribute to the observed resistance in Dielmo and Ndiop.

The complete susceptibility of the mosquito populations to pirimiphos-methyl and bendiocarb underscores the potential utility of these insecticides in managing resistance and controlling malaria transmission. In contrast, resistance to bendiocarb and pirimiphos-methyl has been observed in other regions of Senegal, such as urban Dakar and in the Sudanian zone [19,42].

Interestingly, while the populations were resistant, we observed a significant increase in the mortality rates with deltamethrin in Dielmo between November and December. This difference could be attributed to seasonal variations in mosquito exposure to insecticides or the implementation of effective resistance management strategies, shown to delay resistance onset and restore susceptibility in vector populations [43].

The knockdown times (KDT_50_ and KDT_95_) for the tested pyrethroids revealed a low insecticide action dynamic, with none of the four pyrethroids achieving 100% knockdown after 60 min of exposure. Deltamethrin and permethrin had the shortest KDT_50_ times, indicating a quicker knockdown effect compared to other pyrethroids. However, the overall low knockdown rates reflect the reduced efficacy of these insecticides in the studied populations, indicative of the resistance mechanisms that impair their immediate action [2].

Genotypic analysis of the *L1014F* and *L1014S* mutations in *An. gambiae s.l.* populations revealed interesting patterns. All six genotypes described at the 1014 locus were found in *An. arabiensis* populations, while only four were present in *An. coluzzii* and three in *An. gambiae*. The predominance of the *L1014F* allele across *An. gambiae* and *An. coluzzii* populations and the relatively low frequency of the *L1014S* allele are consistent with other findings documenting the widespread presence of *L1014F* in malaria vectors across Africa [42,44]. This predominance of the *L1014F* mutation within these populations in our study is consistent with prior findings in Senegal [18,45] and elsewhere [46,47]. The geographic variability in the distribution of the *L1014S* mutation, being more prevalent in Ndiop than in Dielmo, underscores the need for localized studies to understand specific resistance profiles in different areas [11,48,49].

The association between *kdr* mutations and pyrethroid resistance showed that the *L1014F* mutation was not significantly correlated with resistance to the tested pyrethroids. This is consistent with previous studies indicating that while *L1014F* contributes to resistance, it may not always be the primary determinant of resistance levels [19,50]. Conversely, the *L1014S* mutation showed significant associations with resistance to lambdacyhalothrin and deltamethrin, particularly in Ndiop. This suggests that the *L1014S* mutation plays a more critical role in conferring resistance to specific pyrethroids in these populations [51,52,53]. The lack of significant correlation between the *L1014S* mutation and resistance to alphacypermethrin and permethrin in Dielmo further highlights the complexity of resistance mechanisms and the possible involvement of other genetic or metabolic factors [54].

Regarding the *N1575Y* mutation, initially reported by Gueye et al. [18] in Senegal, this mutation was exclusively identified in the heterozygous state in Dielmo and was present in only two *An. gambiae* specimens. This mutation has also been reported in other studies in West Africa [15,55]. However, its absence in *An. arabiensis* populations at both sites is consistent with findings from Ethiopia [15,56]. The low frequency of this mutation suggests that it may not yet play a significant role in resistance in the studied populations. Similarly, the absence of the *Ace-1* mutation in the tested specimens indicates that this mutation is not currently contributing to resistance to carbamates and organophosphates in these populations.

Overall, our findings underscore the importance of continuous surveillance and localized strategies to manage insecticide resistance. The complex interplay of different resistance mechanisms, including target-site mutations and metabolic detoxification, necessitates an integrated approach to vector control. Future studies should focus on elucidating the roles of additional genetic mutations and metabolic pathways in resistance and evaluating the effectiveness of alternative insecticides and integrated vector management strategies.

## 5. Conclusions

The high levels of resistance to pyrethroids and DDT in *Anopheles* populations from Dielmo and Ndiop, coupled with the presence of *kdr* mutations, pose significant challenges to malaria control efforts. This study highlights the critical need to assess the expression level of detoxification genes involved in metabolic resistance and to investigate behavioral resistance in vectors for adaptive management strategies. Continuous resistance monitoring is essential to sustain gains in malaria control and work towards malaria elimination. This is particularly important as some species exhibiting resistance to pyrethroids were not carriers of *kdr* or *N1575Y* mutations. A comprehensive understanding of this mechanism is crucial for the design of targeted and sustainable vector control interventions.

## Figures and Tables

**Table 1:** Percentage mortality rates of *An. gambiae s.l.* populations from Dielmo and Ndiop, Senegal, exposed to different insecticides from September to December 2022.

Sites	Insecticides		September		October		November		December
		N	MR (95% CI)	N	MR (95% CI)	N	MR (95% CI)	N	MR (95% CI)
Dielmo	Lamdacyhalothrin (0.05%)	100	85 (76.5–91.4)	100	62 (51.7–75.5)	100	71 (61.1–79.6)	100	31 (22.1–41)
	Alphacypermethrin (0.05%)	-	-	100	62 (51.7–75.5)	-	-	-	-
	Deltamethrin (0.05%)	-	-	-	-	102	52.9 (42.8–62.9)	99	75.7 (66.1–83.8)
	Permethrin (0.75%)	-	-	100	82 (73.1–89.0)	-	-	-	-
	Bendiocarb (0.1%)	100	100 (96.4–100)	-	-	82	100 (95.6–100)	-	-
	Pirimiphos-methyl (0.25%)	100	100 (96.4–100)	-	-	100	100 (96.4–100)	98	100 (96.3–100)
	DDT (4%)	-	-	75	82.7 (72.2–90.4)	-	-	-	-
Ndiop	Lamdacyhalothrin (0.05%)	100	78 (68.6–85.7)	100	57 (46.7–66.9)	-	-	-	-
	Alphacypermethrin (0.05%)	-	-	100	87 (78.8–92.9)	-	-	-	-
	Deltamethrin (0.05%)	-	-	-	-	100	77 (67.5–84.8)	-	-
	Permethrin (0.75%)	-	-	100	88 (80.0–93.6)	-	-	-	-
	Bendiocarb (0.1%)	100	100 (96.4–100)	-	-	-	-	-	-
	Pirimiphos-methyl (0.25%)	100	100 (96.4–100)	-	-	-	-	-	-
	DDT (4%)	-	-	100	85 (76.5–91.4)	-	-	-	-

N: total number tested, MR: mortality rate, CI: confidence interval, -: no test.

**Table 2:** Knockdown times in minutes for *An. gambiae s.l.* populations from Dielmo and Ndiop to different insecticides from September to December 2022.

Sites	KDT	Insecticides			
		Alphacypermethrin (0.05%)	Deltamethrin (0.05%)	Lambdacyhalothrin (0.05%)	Permethrin (0.75%)
Dielmo	KDT_50_ (95% CI)	39 (12–211)	25 (8.86–117)	33 (15–96)	31 (14–88)
	KDT_95_ (95% CI)	98 (27–667)	64 (19–366)	80 (32–263)	78 (31–258)
Ndiop	KDT_50_ (95% CI)	31 (11–140)	25 (11–75)	31 (8.9–216)	28 (9.77–132)
	KDT_95_ (95% CI)	76 (23–428)	58 (23–202)	87 (21–805)	61 (19–340)

**Table 3:** Species composition within *An. gambiae* complex in Dielmo and Ndiop, Senegal, from September to December 2022.

Sites	Mosquito Species				N
	*An. arabiensis*	*An. gambiae*	*An. coluzzii*	Hybrids	
Dielmo	619 (84.9)	81 (11.1)	26 (3.6)	3 (0.4)	729
Ndiop	344 (83.5)	49 (11.9)	14 (3.4)	5 (1.2)	412

N: total number of mosquitoes, (): percentage.

**Table 4:** Genotypic and allelic frequencies of *kdr* mutations in the populations of *An. gambiae* complex species in Dielmo and Ndiop, Senegal, from September to December 2022.

Sites	Species	Genotypes						Allelic Frequencies (%)		
		FF	FS	LF	LL	LS	SS	F	L	S
Dielmo	*An. arabiensis*	2	21	49	98	77	16	0.14	0.61	0.25
	*An. coluzzii*	0	0	12	4	1	0	0.35	0.62	0.03
	*An. gambiae*	0	1	40	4	0	0	0.46	0.53	0.01
Ndiop	*An. arabiensis*	1	16	28	48	50	6	0.15	0.58	0.26
	*An. coluzzii*	0	1	4	4	0	0	0.28	0.67	0.06
	*An. gambiae*	0	0	25	8	0	0	0.38	0.62	0

L: leucine allele (wild type), F: phenylalanine allele (mutant), S: serine allele (mutant), LL: homozygous susceptible, LF: heterozygous *kdr*-West, FF: homozygous resistant *kdr*-West, LS: heterozygous *kdr*-East, SS: homozygous resistant *kdr*-East, FS: heterozygous resistant *kdr*-West and *kdr*-East.

**Table 5:** Genotypic and allelic frequencies of the *N1575Y* mutation in the populations of *An. gambiae* complex species in Dielmo and Ndiop, Senegal, from September to December 2022.

Sites	Species	Genotypes				Allelic Frequencies	
		Nb	NN	NY	YY	N	Y
Dielmo	*An. arabiensis*	259	259	0	0	100	0
	*An. gambiae*	45	43	2	0	98	2
	*An. coluzzii*	16	16	0	0	100	0
Ndiop	*An. arabiensis*	148	148	0	0	100	0
	*An. gambiae*	33	33	0	0	100	0
	*An. coluzzii*	9	9	0	0	100	0

Nb: number of specimens, Y: mutant-resistant allele (tyrosine), N: wild susceptible allele (aspargine), NN: homozygous susceptible, NY: heterozygous, YY: homozygous resistant.

**Table 6:** Association between the *L1014F* mutation and pyrethroid resistance in the populations of *An. gambiae* complex species in Dielmo and Ndiop, Senegal, from September to December 2022.

Sites	Insecticides	Status	*L1014F*	*L1014L*	Total	Freq	OR	95% CI	*p*-Value
Dielmo	Alphacypermethrin (0.05%)	Survivors	14	40	54	26	1		
		Dead	17	41	58	29	1.18	0.52–2.72	0.69
	Deltamethrin (0.05%)	Survivors	15	83	98	15	1		
		Dead	10	90	100	10	0.61	0.26–1.44	0.26
	Lambdacyhalothrin (0.05%)	Survivors	23	77	100	23	1		
		Dead	29	107	136	21	0.9	0.49–1.69	0.76
	Permethrin (0.75%)	Survivors	10	26	36	28	1		
		Dead	9	51	60	15	0.46	0.17–1.27	0.13
Ndiop	Alphacypermethrin (0.05%)	Survivors	5	19	24	21	1		
		Dead	9	51	60	15	0.67	0.20–2.26	0.51
	Deltamethrin (0.05%)	Survivors	12	34	46	26	1		
		Dead	5	37	42	12	0.38	0.12–1.20	0.09
	Lambdacyhalothrin (0.05%)	Survivors	13	45	58	22	1		
		Dead	21	47	68	31	1.55	0.69–3.45	0.29
	Permethrin (0.75%)	Survivors	5	19	24	21	1		
		Dead	8	54	62	13	0.56	0.16–1.93	0.36

*L1014F*: mutation *kdr-west*, *L1014L*: wild type; Freq: allelic frequencies as a percentage, OR: odds ratio.

**Table 7:** Correlation between the *L1014S* mutation and pyrethroid resistance in the populations of *An. gambiae* complex species in Dielmo and Ndiop, Senegal, from September to December 2022.

Sites	Insecticides	Statuts	*L1014S*	*L1014L*	Total	Freq	OR	95% CI	*p*-Value
Dielmo	Alphacypermethrin (0.05%)	Survivors	15	39	54	28	1		
		Dead	8	52	60	13	0.4	0.15–1.04	0.06
	Deltamethrin (0.05%)	Survivors	29	69	98	30	1		
		Dead	18	82	100	18	0.52	0.27–1.02	0.06
	Lambdacyhalothrin (0.05%)	Survivors	29	73	102	28	1		
		Dead	14	126	140	10	0.28	0.14–0.56	0.0002
	Permethrin (0.75%)	Survivors	11	25	36	31	1		
		Dead	9	51	60	15	0.4	0.15–1.09	0.07
Ndiop	Alphacypermethrin (0.05%)	Survivors	7	17	24	29	1		
		Dead	7	53	60	12	0.32	0.09–1.04	0.05
	Deltamethrin (0.05%)	Survivors	18	28	46	39	1		
		Dead	7	33	40	18	0.33	0.12–0.90	0.03
	Lambdacyhalothrin (0.05%)	Survivors	17	41	58	29	1		
		Dead	8	62	70	11	0.31	0.12–0.79	0.01
	Permethrin (0.75%)	Survivors	7	17	24	29	1		
		Dead	11	51	62	18	0.52	0.17–1.56	0.24

*L1014S*: mutation *kdr-East*, *L1014L:* wild type, Freq: allelic frequencies as a percentage, OR: odds ratio.

## Data Availability

The data for this study have been presented within this article, and any further information regarding this study can be reasonably requested from the corresponding author.
